# Primary, Secondary Metabolites, Photosynthetic Capacity and Antioxidant Activity of the Malaysian Herb Kacip Fatimah (*Labisia Pumila* Benth) Exposed to Potassium Fertilization under Greenhouse Conditions

**DOI:** 10.3390/ijms131115321

**Published:** 2012-11-20

**Authors:** Mohd Hafiz Ibrahim, Hawa Z. E. Jaafar, Ehsan Karimi, Ali Ghasemzadeh

**Affiliations:** Department of Crop Science, Faculty of Agriculture, University Putra Malaysia, 43400 Serdang, Selangor, Malaysia

**Keywords:** *Labisia pumila*, potassium fertilization, primary and secondary metabolites, leaf gas exchange, antioxidant enzyme activity

## Abstract

A randomized complete block design was used to characterize the relationship between production of total phenolics, flavonoids, ascorbic acid, carbohydrate content, leaf gas exchange, phenylalanine ammonia-lyase (PAL), soluble protein, invertase and antioxidant enzyme activities (ascorbate peroxidase (APX), catalase (CAT) and superoxide dismutase (SOD) in *Labisia pumila* Benth var. *alata* under four levels of potassium fertilization experiments (0, 90, 180 and 270 kg K/ha) conducted for 12 weeks. It was found that the production of total phenolics, flavonoids, ascorbic acid and carbohydrate content was affected by the interaction between potassium fertilization and plant parts. As the potassium fertilization levels increased from 0 to 270 kg K/ha, the production of soluble protein and PAL activity increased steadily. At the highest potassium fertilization (270 kg K/ha) *L. pumila* exhibited significantly higher net photosynthesis (A), stomatal conductance (g_s_), intercellular CO_2_ (C_i_), apparent quantum yield (ξ) and lower dark respiration rates (R_d_), compared to the other treatments. It was found that the production of total phenolics, flavonoids and ascorbic acid are also higher under 270 kg K/ha compared to 180, 90 and 0 kg K/ha. Furthermore, from the present study, the invertase activity was also found to be higher in 270 kg K/ha treatment. The antioxidant enzyme activities (APX, CAT and SOD) were lower under high potassium fertilization (270 kg K/ha) and have a significant negative correlation with total phenolics and flavonoid production. From this study, it was observed that the up-regulation of leaf gas exchange and downregulation of APX, CAT and SOD activities under high supplementation of potassium fertilizer enhanced the carbohydrate content that simultaneously increased the production of *L. pumila* secondary metabolites, thus increasing the health promoting effects of this plant.

## 1. Introduction

A lot of research in recent years has been paying attention to phenolic acid and flavonoid intake from the human diet and possible health benefits due to the antioxidant nature of the aromatic phenolics and flavonoid structures [1,2]. Phenolic acids and flavonoids are believed to be responsible for the wide spectrum of pharmacological activities seen in many plants [3]. Phenolic acids, including gallic acid, benzoic acids and cinnamic acids, constitute a major group of plant secondary metabolites. Nowadays, phenolic acids are receiving considerable attention because of their reported protective role against cancer and heart disease. This role may be attributed to their antioxidant activity against reactive oxygen species, which is reported to be higher than that of vitamins C and E [4]. Flavonoids are polyphenolic compounds that contain a C15 flavone skeleton and consists of the flavones, flavonols, flavanols, flavanone and flavanonols, which together represent the majority of plant secondary metabolites. These components are thought to play a role in the protection of plants from pests and diseases. Moreover, flavonoids have remarkable health promoting effects, such as anti-inflammatory [5], anti-microbial [6], antioxidant [7], anti cancer activity [8] as well as the prevention of osteoporosis [9].

One plant that contains high levels of phenolics acid and flavonoids is *Labisia pumila*. also known locally in Malaysia as Kacip fatimah. Both phenolic acids and flavonoids are believed to be responsible for the wide spectrum of pharmacological activities attributed to this herb. *Labisia pumila* that has been widely applied as a decoction in South East Asian communities for a variety of illnesses and also used in health supplements [10]. The water decoction of *L. pumila* is traditionally consumed by ethnic Malay women to treat menstrual irregularities and painful menstruations, as a postpartum remedy for toning vaginal walls, to generally alleviate fatigue and to promote emotional well-being [11]. Traditionally, *L. pumila* extract is prepared by boiling the roots, leaves or the whole plant in water and the extract is taken orally and used to accelerate labor, shrink the uterus, improve menstrual cycle and for weight loss [12]. The antioxidant activity of the aqueous *L. pumila* extract has been reported as providing significant protection to human dermal fibroblasts and from cell damage caused by UV irradiation [13], most likely due to the presence of secondary metabolites, *i.e.*, flavonoids and phenolics [14].

The levels and composition of phenolics acid and flavonoids in plants varies according to genotype, climate factors such as seasonal variation, light intensity, relative humidity and temperature, environment stimuli and agronomical practices [15–18]. Cultivation factors such as soil type, compost, mulching and fertilization also can affect the plant secondary metabolites and antioxidant activity of plant. In all these factors research are uncovering the fact that the availability of plant nutrients are the most important factors in determining secondary metabolism and antioxidant activity within plants [19,20]. Potassium as macronutrient is one of the most important nutrients in controlling yield and quality of plants [21–23]. Potassium is a mineral nutrient of specific importance in developing tissues and plays important role in osmotic regulation and phloem transportation [24]. Potassium also is required for the activation of certain enzyme in extracts of higher plants, and K deficient plants usually have lower activities of these enzymes in their extracts than normal plants [25].

Despite enzyme activation the levels and composition of primary and secondary compounds are also determined with K supplementation to the plant. Primary metabolites (starch and soluble sugar) usually decrease in K deficiency situations, as reported in alfalfa [26], sugar beet [27], cotton [28], soybean [29] and wheat [30]. Primary metabolite accumulation in plants with adequate K has been explained by a requirement for K in phloem transportation [31]. Besides carbohydrates, amino acid accumulation under excessive K fertilization was also reported in tobacco [32], rice and barley [33]. As carbohydrate decreased under K deficiency it was also reported that secondary metabolites were also influenced by K application. Liaqat *et al*. [34] observed total phenolics in blackberries decreased as potassium levels decreased. Lehman and Rice [35] found scopolin content reduced as K levels decreased in sunflower. Troufflard *et al.*[36] also found the content of oxylipins in *Arabidopsis thaliana* was reduced in K deficient plants. Under K deficiency, Lubbe *et al.*[37] reported that the production of galanthamine in *Narcissus* bulbs was reduced. These results all suggest the importance of K in regulating the production of secondary metabolites in plants.

Previous studies on *L. pumila* performed with different nitrogen fertilization regimes have shown that high nitrogen can reduce the production of secondary metabolites in this herb due to reduced phenyl alanine ammonia-lyase (PAL) activity that was correlated with low C/N ratio, photosynthetic rates and total non structural carbohydrate (TNC) [38]. However, no documentation of the phytochemical responses of *L. pumila* to other nutrients, especially potassium, has been reported [39]. This information is important and will be useful in the cultivation of this plant. Usually, plants fertilized with high potassium levels tend to increase their carbohydrate and bioactive compound levels [40,41]. Many studies have investigated the effects of potassium fertilization on the vegetative and yield aspects, but relatively few studies have investigated the response of plant secondary metabolites to increasing potassium fertilization, particularly on the medicinal value of local Malaysian herb *Labisia pumila*[42]. The objective of this study was to examine the effects of different potassium levels on primary (total non-structural carbohydrate), and secondary (total flavonoids and total phenolics) metabolites, leaf gas exchange characteristics, soluble protein, antioxidant enzyme and PAL activity *L. pumila* var *alata*. Relationships among the parameters were also determined to characterize their cross involvement.

## 2. Results and Discussion

### 2.1. Total Phenolics and Flavonoids Profiling

Accumulation of total phenolics and flavonoids in *L. pumila* were influenced by potassium levels (*p* ≤ 0.01; Table 1). Generally total phenolics content was highest in the leaves followed by stems and roots. As the plant receives high K levels (0 > 270 kg K/ha) the production of total phenolics and flavonoids was enhanced. The total phenolics content in leaf-180 kg K/ha, leaf-90 kg K/ha, leaf-0 kg K/ha, stem-270 kg K/ha, stem-180 kg K/ha, stem-90 kg K/ha, stem-0 kg K/ha, root-270 kg K/ha, root-180 kg K/ha, root-90 kg K/ha and root-0 kg K/ha were 10, 23, 35, 47, 52, 61, 65, 66, 68, 77 and 78%, respectively, were low compared to the leaf at 270 kg K/ha that registered 1.82 mg gallic acid g^−1^ dry weight. Total flavonoids content followed the same trend as total phenolics where the highest total flavonoids was observed in leaf at 270 kg K/ha that registered 0.94 mg rutin g^−1^ dry weight and the lowest was in the root at 0 kg K/ha that contained only 0.18 mg rutin g^−1^ dry weight.

The increase in the production of total phenolics and flavonoids with increasing potassium levels in the study might be due to enhancement of total non-structural carbohydrate (TNC) [43,44]. This might due to potassium’s role of stimulating photosynthesis activity and increasing the translocation of carbohydrate to plant parts [45,46]. The increase in translocation indirectly enhanced the biosynthesis of total phenolics and flavonoids of *L. pumila* treated with high potassium fertilizer. From correlation Table 2 it was shown that TNC had a high significant positive correlation with total phenolics (*R*^2^ = 0.985; *p* ≤ 0.05) and total flavonoids (*R*^2^ = 0.812; *p* ≤ 0.05) that indicate the increase of production of total phenolics and flavonoids under high potassium levels might be due to increase availability of TNC [47,48]. The present findings was also in agreement with Wei *et al.*[8] that found the increase in total phenolics and flavonoids in *Chrysanthemum morifolium* leaves was due to increase in production of TNC under high K application. This implies that an increasing potassium supply can enhance production of TNC and simultaneously increase the production of plant secondary metabolites in *L. pumila* seedlings.

### 2.2. Ascorbic Acid and Their Profiling

Ascorbic acid, also known as vitamin C, is one of the most abundant antioxidants in plant where the role of ascorbate is to protect plants against oxidative stress [49]. It is a powerful water soluble antioxidant and its established role is to prevent scurvy [50]. The profiling of ascorbic acid in *L. pumila* plants followed the same trend as the total phenolics and flavonoids, where the availability of ascorbic acid was found to be higher in the leaves followed by stems and lowest in roots (Table 1). The imposition of high K levels has resulted in significantly higher ascorbic acid contents in the leaves, stems and roots of *L. pumila.* By the end of week 15 after start of treatments, the ascorbic acid contents in the leaves of plants receiving 0, 90 and 180 kg K/ha were 0.030, 0.047 and 0.062 mg g^−1^l-ascorbic acid fresh weight, respectively, compared to 0.083 mg g^−1^l-ascorbic acid fresh weight achieved with 270 kg K/ha application. The same observation was made by Liaqat *et al*. [34] and Prasad and Spiers [51] when they observed ascorbic acid content in blackberries and Kiwi fruit was substantially enhanced with application of high amounts of K fertilizer. The increase in ascorbic acid content under high K application levels in *L. pumila* seedlings might possibly be attributed to high production of TNC under high K application. This is because TNC (d-glucose) is a precursor for ascorbic acid biosynthesis in plants, as more availability of TNC more ascorbic acid would be produced in the l-galactose pathways [52,53]. This fact was supported by positive relationship of ascorbic acid with TNC (*R*^2^ = 0.765; *p* ≤ 0.05; Table 2). The present result indicated that accumulation of TNC in high fertilized K plant would increase the production of ascorbic acid in *L. pumila*.

### 2.3. Total Soluble Sugar, Starch and Total Non-Structural Carbohydrate (TNC) and Their Profiling

Primary metabolites also followed the same patterns as total phenolics, flavonoids and vitamin C. The accumulation of soluble sugar, starch and TNC was influenced by K levels (*p* ≤ 0.05; Table 3). The highest accumulation of primary metabolites was also highest in leaves and lowest in the roots. The soluble sugar content in leaf-270 kg K/ha, leaf-180 kg K/ha, leaf-90 kg K/ha, leaf-0 kg K/ha, stem-270 kg K/ha, stem-180 kg K/ha, stem-90 kg K/ha, stem-0 kg K/ha, root-270 kg K/ha, root-180 kg K/ha, root-90 kg K/ha and root -0 kg K/ha were 79, 69, 60, 46, 41, 28, 26, 18 and 14%, respectively, higher compared to the root at 0 kg K/ha that only registered 29.61 mg g^−1^ dry weight. The starch and TNC showed similar patterns as soluble sugar. Previous reports showed there was less accumulation of soluble sugar and increase in starch content under high K fertilization [29,40,41]. In the present results it was shown as the K increased the soluble sugar and starch content tended to accumulate. The differences in the carbohydrate accumulation in the study with the previous study might be due to the sink strength of the plant to K fertilization response [54]. Usually, plants with high sink strength would accumulate more starch and soluble sugar in their plant parts. In the present study, it was observed that there was more accumulation of starch than soluble sugar in *L. pumila* plants fertilized with high K without impairment of photosynthesis. Usually, plant with high sink strength would accumulate more starch and less soluble sugar with no impairment of photosynthesis under high potassium fertilization [29,55]. This indicate that *L. pumila* have high sink strength under high supplementation of K, however the response are different reported in other plant due to differences in the plant species, the plant sink strength and the sources of K that been used [26–28,56–58]. In the present study, the excess in TNC under high K fertilization would be used for growth and production of secondary metabolites [42]. The increase in production of TNC under excess K fertilization was also observed by Pettigrew [22] and Liu *et al*. [58] in cotton and *Chrysanthemum morifolium*. The increase in carbohydrate production under high K fertilization might be due to increase in starch synthetase (EC 2.4.1.21) activity that enhanced the production of carbohydrate. This is because starch synthetase activity is activated by K, thus with adequate K the levels of starch and soluble sugar accumulated [59–61]. From the correlations in Table 2, soluble sugar, starch and TNC were observed to have strong significant positive correlations with total phenolics and flavonoids. Carbohydrates are basic compounds required to produce phenolic compounds through the shikimic acid pathway where extra carbohydrates derived from glycolysis and the pentose phosphate pathway are converted into aromatic amino acids [62]. Previous studies by Shui *et al.*[62] showed that an increase in secondary metabolites was related to the balance between carbohydrate source and sink; the greater the source-sink ratio, the greater the production of secondary metabolites that might occur.

### 2.4. Phenylalanine-Ammonia-Lyase; PAL Activity and Soluble Protein

The PAL activity was influenced by potassium levels (*p* ≤ 0.05; Table 4). The PAL activity was found to be highest under 270 kg K/ha (37.28 nM transcinnamic mg^−1^ protein^−1^ hour^−1^) followed by 180 kg K/ha (25.61 nM transcinnamic mg^−1^ protein^−1^ hour^−1^), 90 kg K/ha (19.28 nM transcinnamic mg^−1^ protein^−1^ hour^−1^) and the lowest at 0 kg K/ha that just recorded 8.24 nM transcinnamic mg^−1^ protein^−1^ hour^−1^. The soluble protein content followed the same trend as PAL activity. As potassium fertilization increases from 90 > 180 > 270 kg K/ha soluble protein increased by 180%, 495% and 958%, respectively, compared to control (0 kg K/ha) that just recorded 1.17 mg/g fresh weight protein. The increase in production of secondary metabolites in the present work could be due to increase in PAL activities under high K fertilization. Correlation analysis (Table 2) showed that PAL had a significant positive relationship with total phenolics and flavonoids (*R*^2^ = 0.945; *R*^2^ = 0.987; *p* ≤ 0.05), which might indicate an upregulation of plant secondary metabolite production with increased PAL activity. This is basically due to the fact that PAL is a precursor to total phenolics and flavonoids biosynthesis [63]. The increase in PAL activity under high K fertilization was also observed by Wei *et al*. [8] in *Chrysanthemum morifolium*. Reasons can be higher PAL gene expression which was observed by Li *et al.*[63] in maize when exposed to high K levels or general enzyme activation by potassium. This also might be due the role of potassium in enzyme activation. The increase in total phenolics and flavonoid biosynthesis with increasing potassium fertilization might be due to the role of potassium as a cofactor in phenylalanine ammonia-lyase (PAL) enzyme activity. With more potassium available, the activity of PAL enzyme increases and more biosynthesis of secondary metabolites might occur [64,65]. This was observed by Devi *et al*. [66] where a high supply of potassium increased the PAL and antioxidant activity in *Panax ginseng*.

### 2.5. Leaf Gas Exchange Properties

For this study, the net photosynthetic rate (A), stomatal conductance (g_s_), intercellular CO_2_ (C_i_), dark respiration rates (R_d_) and apparent quantum yield (ξ) were determined by a portable infrared photosynthesis system LI-6400 (LI-COR, Lincoln, NE, USA). In general, the photosynthesis rate (A) was found to be higher in 270 kg K/ha (12.72 μmol m^−2^ s^−1^), followed by 180 kg K/ha (8.45 μmol m^−2^ s^−1^), 90 kg K/ha (6.75 μmol m^−2^ s^−1^) and lowest in 0 kg K/ha that just recorded 3.45 μmol m^−2^ s^−1^ (Table 5). The same pattern was followed with stomatal conductance (g_s_) and intercellular CO_2_ (C_i_). It was found that g_s_ and C_i_ have a significant positive correlation with net photosynthesis (*R*^2^ = 0.987; *R*^2^ = 0.908; *p* ≤ 0.01; Table 2) respectively, this indicate that the increase in photosynthesis rate under high K supplementation might be due increase in g_s_ that was stimulated with high K that simultaneously enhanced the C_i_ inside the leaves [67,68]. A previous study by Peoples and Coach [69] had shown that increased K fertilization can enhance the conductance in *Medicago sativa* and simultaneously enhance the uptake of CO_2_ that increases C_i_ levels. The role of K in photosynthesis is complex. The activation of enzymes by K and its involvement in adenosine triphosphate (ATP) production is more important in regulating the rate of photosynthesis than the role of K in stomatal conductance [70]. This is because when plant are lacking in K, the rate of photosynthesis and the rate of ATP production are reduced, and all the process reliant on ATP are slowed down [71]. Conversely, plant respiration rate increases, which also contributes to slower growth and development. This phenomenon was shown in the present study where *L. pumila* fertilized with 270, 180 and 90 kg K/ha had lower respiration rates (2.14, 4.11, 6.23 μmol m^−2^ s^−1^), respectively, compared to control plants (0 kg K/ha) that registered the highest respiration rate (8.24 μmol m^−2^ s^−1^). Furthermore, the plant treated with high potassium fertilization have shown enhanced light harvesting efficiency by having a high apparent quantum yield. At 270 kg K/ha, the apparent quantum yield of *L. pumila* was 0.097 μmol m^−2^ s^−1^, compared to 180, 90 and 0 kg K/ha that recorded 0.078, 0.043 and 0.032 μmol m^−2^ s^−1^, respectively. According to Lam *et al.*[72] the increase in net photosynthesis with increase K might be due to increase in nitrate reductase activity that directly involved in photosynthetic enzyme regulation. From correlation Table 2 it were found that A have a significant positive correlationship with total phenolics (*R*^2^ = 0.998; *p* ≤ 0.05) and total flavonoid (*R*^2^ = 0.897 *p* ≤ 0.05) that indicate an increase in production of secondary metabolites under high K fertilization might be due to increase photosynthetic capacity of *L. pumila* that stimulated the shikimic acid pathway. [73]. This suggest the importance of K in regulation of leaf gas exchange and secondary metabolites in *L. pumila*. [74–76].

### 2.6. Leaf Invertase Activity

**Figure 1:**
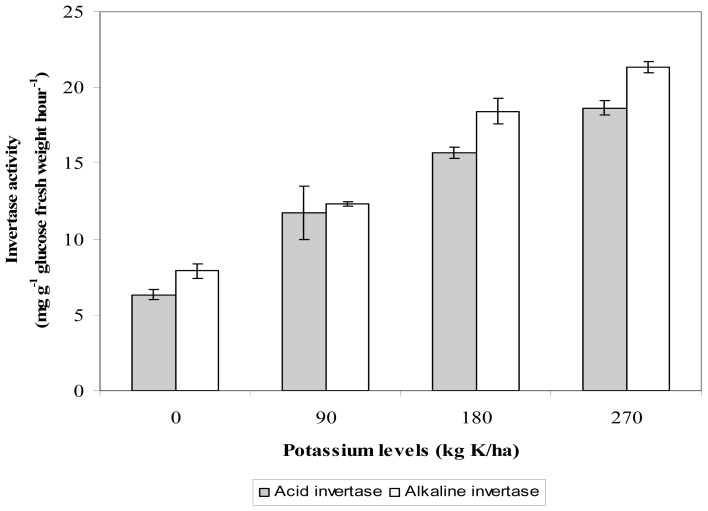
The effects of different potassium fertilization on invertase activity in leaves of *L. pumila. N* = 40. Bars represent standard error of differences between means (SEM).

The leaf invertase activity was influenced by K levels applied (*p* ≤ 0.05; Figure 1). Leaf acid invertase and alkaline invertase increased with increasing K fertilization in an ascending order 0 < 90 < 180 < 270 kg K/ha. The highest acid and alkaline invertase activity was obtained in *L. pumila* fertilized with 270 kg K/ha (18.65 and 21.335 mg g^−1^ glucose fresh weight hour^−1^), respectively, compared to control (0 kg K/ha), that recorded 6.32 mg g^−1^ glucose fresh weight hour^−1^ for acid invertase and 7.89 mg g^−1^ glucose fresh weight hour^−1^ for alkaline invertase. Invertase can be considered a key enzyme in carbohydrate metabolism since it catalyzes the irreversible reaction that converts sucrose into glucose and fructose [77]. Potassium plays a critical role in phloem translocation mechanism. The increase in invertase activity showed enhanced phloem translocation mechanism under high K fertilization [78]. This is because an increase in invertase activity would increase the conversion of sucrose to glucose and fructose. Higher glucose levels with more potassium supply would produce more ATP in the citric acid cycle that will be used in phloem translocation of nitrates, minerals and amino acids [79]. According to Lei and Yan [77] the enhanced invertase activity correspond to enhanced malondialdehyde and nitrate reductase in *Stevia rebaudiana*. From correlation Table 2 it was found that acid and alkaline invertase activity have a significant positive correlation with total phenolics and flavonoids that indicated the increase in conversion of sucrose to glucose and fructose in high invertase activity might increase the availability of glucose that might enhanced production of plant secondary metabolites (total phenolics, flavonoid and ascorbic acid). The present finding showed the importance of K in enhancing the availability of glucose by promoting high invertase activity.

### 2.7. Antioxidant Enzyme Activities

Activities of the antioxidant enzymes ascorbate peroxidase (APX), catalase (CAT) and superoxide dismutase (SOD) were significantly (*p* ≤ 0.05) affected by potassium fertilization (Table 6). The APX, CAT and SOD activities were found lowest at maximum potassium fertilization at 270 kg P/ha. These activities are an indication that enhanced potassium fertilization can reduce the oxidative stress to *L. pumila* seedlings. Under stress potassium plays an important role in the synthesis of protein by participating in polypeptide synthesis in ribosomes, since that process requires a high concentration of potassium [80]. It was reported by Tripathi *et al.*[81] that proteins such as thioredoxin, glutaredoxin and cyclophilin are known to facilitate the regeneration of the reduced (catalytically active) form of peroxyredoxin that plays an important role in reducing the ROS formation in plants under biotic and abiotic stress. The reduction in APX, CAT and SOD activities have shown to have a negative significant correlation with production of total phenolics and flavonoids that indicate the impairment of oxidative stress can enhance the secondary metabolites of *L. pumila* seedlings under high potassium fertilization. Generation of ROS, particularly H_2_O_2_, had been proposed to be part of the signaling cascades that lead to protection from stress. Induction of antioxidant enzymes was reported to be a general strategy adopted by plants to overcome oxidative stresses. The APX, CAT and SOD function as effective quenchers for ROS [82]. CAT plays an essential role in scavenging from H_2_O_2_ toxicity. The combined action of CAT and SOD converts the O^2−^ and H_2_O_2_ to water and molecular oxygen (O_2_), thus prevent the cellular damage under unfavorable condition [83]. In the present study potassium plays a key role in reduction of ROS production by reducing activity of NAD(P)H oxidase and maintaining electron transport [84]. The present result indicated that if oxidative stress can be reduced by increasing potassium fertilization, the production of secondary metabolites in *L. pumila* can be enhanced. These result also implied that fertilization with high potassium can play important protective role in O^2−^ and H_2_O_2_ scavenging processes. In correlation Table 2 it is shown that APX, CAT and SOD have a significant negative correlation with total phenolics and flavonoids (*p* ≤ 0.05). This indicates that the antioxidant activity and secondary metabolite levels that indicate up-regulation of secondary metabolites might be occurring under low oxidative stress with high potassium fertilization in *L. pumila* benth seedlings.

## 3. Experimental

### 3.1. Experimental Location, Plant Materials and Treatments

The experiment was carried out in growth houses at Field 2, Faculty of Agriculture Greenhouse Complex, Universiti Putra Malaysia (longitude 101°44′ N and latitude 2°58′ S, 68 m above sea level) with a mean atmospheric pressure of 1.013 kPa. Three-month old *L. pumila* seedlings of var. *alata*, were left for a month to acclimatize in a nursery until ready for the treatments, then they were fertilized with four rates of potassium applied in the form of muriate of potash (MOP), *viz*. 0 kg K/ha (0.0 g per plant), 90 kg K/ha (0.25 g per plant), 180 kg K/ha (0.51 g per plant) and 270 kg K/ha (0.76 g per plant). The potassium was split into three fertilization phases, and each phase was about 33.3% of total potassium fertilizer. Every potassium treatment received urea (46% N; 0.72 g per plant) and Triple Super Phosphate, TSP (60% K; 0.51 g per plant) at standard rates of 180 kg /ha during the studies there were no indication of K deficiency in all the plant in 0 kg K/ha. The seedlings were planted in soilless medium containing coco-peat and well composted chicken manure in 5:1 (*v*/*v*) ratio in 25 cm diameter polyethylene bags. The medium properties are presented in Table 7. Day and night temperatures in the greenhouse were maintained at 27–30 °C and 18–21 °C, respectively, and relative humidity from 50% to 60%. All the seedlings were irrigated using overhead mist irrigation given four times a day or when necessary. Each irrigation session lasted for 7 min [85]. The experiment was based on a Randomized Complete Block Design (RCBD) with four replicates. The factor was four levels of potassium fertilization (0, 90, 180 and 270 kg K/ha). Each combination treatment consisted of 10 plants totaling a sum of 160 plants used in the experiment. Plants were harvested at 12 weeks after planting.

### 3.2. Total Phenolics and Flavonoids Quantification

The method of extraction and quantification for total phenolics and flavonoids contents followed after Ibrahim *et al*. [86]. An amount of ground dried tissue samples (0.1 g) was extracted with 80% ethanol (10 mL) on an orbital shaker for 120 min at 50 °C. The mixture was subsequently filtered (Whatman™ No.1), and the filtrate was used for the quantification of total phenolics and total flavonoids. Folin-Ciocalteu reagent (diluted 10-fold) was used to determine the total phenolics content of the leaf samples. The sample extract (200 μL) was mixed with Folin-Ciocalteau reagent (1.5 mL) and allowed to stand at 22 °C for 5 min before adding NaNO_3_ solution (1.5 mL, 60 g L^−1^). After two hours at 22 °C, absorbance was measured at 725 nm. The results were expressed as mg g^−1^ gallic acid equivalent (mg GAE g^−1^ dry sample). For total flavonoids determination, a sample (1 mL) was mixed with NaNO_3_ (0.3 mL) in a test tube covered with aluminium foil, and left for 5 min. Then 10% AlCl_3_ (0.3 mL) was added followed by addition of 1 M NaOH (2 mL). Later, the absorbance was measured at 510 nm using a spectrophotometer with rutin as a standard (results expressed as mg g^−1^ rutin dry sample).

### 3.3. Ascorbic Acid Content

The ascorbic acid content was measured using a modified method of Davis and Masten [87]. The fresh leaf samples (1 g) were extracted in 1% of phosphate-citrate buffer (2 mL, pH 3.5) using a chilled pestle and mortar. The homogenate was filtered. The filtrate was added to the 1 mL of 1.7 mM 2,6-dichloroindophenol (2,6-DCPIP) in a 3 mL cuvette. The absorbance at 520 nm was read within 10 min of mixing the reagents. The extraction buffer was used as a blank. l-Ascorbic acid was used as a standard. Ascorbic acid was recorded as mg g^−1^l-ascorbic acid fresh leaves

### 3.4. Total Soluble Sugar Determination

Total soluble sugar was measured spectrophotometrically using the method of Ibrahim and Jaafar [88] Samples (0.5 g) were placed in 15 mL conical tubes, and distilled water added to make up the volume to 10 mL. The mixture was then vortexed and later incubated for 10 min. Anthrone reagent was prepared using anthrone (Sigma Aldrich, St. Louis, MO, USA, 0.1 g) that was dissolved in 95% sulphuric acid (Fisher Scientific, Chicago, IL, USA, 50 mL). Sucrose was used as a standard stock solution to prepare a standard curve for the quantification of sucrose in the sample. The mixed sample of ground dry sample and distilled water was centrifuged at a speed of 3400 rpm for 10 min and then filtered to get the supernatant. A sample (4 mL) was mixed with anthrone reagent (8 mL) and then placed in a water-bath set at 100 °C for 5 min before the sample was measured at an absorbance of 620 nm using a spectrophotometer model UV160U (Shimadzu Scientific, Kyoto, Japan). The total soluble sugar in the sample was expressed as mg sucrose g^−1^ dry sample.

### 3.5. Starch Determination

Starch content was determined spectrophotometrically using a method described by Thayumanavam and Sadasivam [89]. In this method, dry sample (about 0.5 g) was homogenized in hot 80% ethanol to remove the sugar. The sample was then centrifuged at 5000 rpm for 5 min and the residue retained. After that, distilled water (5.0 mL) and 52% perchloric acid (6.5 mL) were added to the residue. Then the solution was centrifuged and the supernatant separated and then filtered with Whatman No. 5 filter paper. The processes were repeated until the supernatant was made up to 100 mL. A sample (100 μL) of the supernatant was added to distilled water until the volume became 1 mL in a test tube. After that, anthrone reagent (4 mL, prepared with 95% sulphuric acid) was added to the test tube. The mixed solution was placed in the water bath at 100 °C for eight min and then cooled to room temperature, and then the sample was read at absorbance of 630 nm to determine the sample starch content. Glucose was used as a standard and starch content was expressed as mg glucose equivalent g^−1^ dry sample.

### 3.6. Total Non-Structural Carbohydrate (TNC)

The total non-structural carbohydrate was calculated as the sum of total soluble sugar and starch content [90].

### 3.7. Phenylalanine Ammonia-Lyase (PAL)

Phenylalanine-ammonia-lyase (PAL) activity was measured using the method described by Martinez and Lafuante [91]. The enzyme activity was determined by measuring spectrophotometrically the production of trans-cinnamic acid from l-phenylalanine. Enzyme extract (10 μL) was incubated at 40 °C with 12.1 mM l-phenylalanine (90 μL, Sigma) that were prepared in 50 mM Tris-HCl, (pH 8.5). After 15 min of reaction, trans-cinnamic acid yield was estimated by measuring increase in the absorbance at 290 nm. Standard curve was prepared by using a trans-cinnamic acid standard (Sigma) and the PAL activity was expressed as nM trans-cinnamic acidμg protein^−1^ h^−1^.

### 3.8. Protein Determination

Protein content was determined using the method of Ibrahim and Jaafar [92]. In this method, fresh leaf samples (about 2 g) were cut into pieces using scissors and ground in mortar with 0.05 M Tris buffer (1 mL, pH 8.5) and powdered with liquid nitrogen. The homogenate was then centrifuged at 9000 rpm for 10 min and then stored under refrigeration at 4 °C for 24 h. After the extraction, supernatant from the samples (about 100 μL) was added to Bradford reagent (3 mL, Sigma, prepared using 10 mL of the reagent diluted with 50 mL distilled water) and then incubated for 5 min before being measured at 595 nm with the spectrophotometer. In this method bovine serum (Sigma) was used as a standard to produce calibration curve between actual protein content and spectrophotometer readings. The protein was expressed as mg g^−1^ protein fresh weight.

### 3.9. Leaf Gas Exchange Measurement

The measurement was obtained from a closed infra-red gas analyzer LICOR 6400 Portable Photosynthesis System (IRGA, Licor. Inc. Nebraska, NE, USA). Prior to use, the instrument was warmed for 30 min and calibrated with the ZERO IRGA mode. Two steps are required in the calibration process: first, the initial zeroing process for the built-in flow meter; and second, zeroing process for the infra-red gas analyzer. The measurements used optimal conditions set of 400 μmol mol^−1^ CO_2_ 30 °C cuvette temperature, 60% relative humidity with air flow rate set at 500 cm^3^ min^−1^, and modified cuvette condition of 800 μmol m^−2^ s^−1^ photosynthetically photon flux density (PPFD). The measurements of gas exchange were carried out between 09:00 and 11:00 a.m. using fully expanded young leaves numbered three and four from plant apex to record net photosynthesis rate (A). The operation was automatic and the data were stored in the LI-6400 console and analyzed by “*Photosyn Assistant*” software (Version 3, Lincoln Inc., Columbus, OH, USA). Several precautions were taken to avoid errors during measurements. Leaf surfaces were cleaned and dried using tissue paper before enclosed in the leaf cuvette [93]. The light response curve was produced followed procedures from Ibrahim and Jaafar [94] to generate the apparent quantum yield and dark respiration rate.

### 3.10. Invertase Determination

Invertases were assayed according to Schaffer *et al.*[95] with some modifications. Leaf tissue (2.0 g fresh weight) was extracted in buffer (10 mL) containing 50 mM HEPES-NaOH (pH 7.5), 5 mM MgCl_2_, 1 mM Na_2_EDTA, and 0.05% Triton X-100. The extract was passed through Microcloth and centrifuged at 17,300× *g* for 20 min at 4 °C. The supernatant was concentrated by the addition of (NH_4_)_2_SO_4_ to 80% saturation. After centrifugation for 15 min at 20,000 rpm the precipitate was resuspended in 2 mL of extraction buffer and dialyzed overnight against 5 mM HEPES-NaOH (pH 7.5), 5 mM MgCl_2_, and 1 mM Na_2_EDTA. The invertase assay mixture contained 200 μL dialyzed extract, 200 mM sucrose, and 100 mM citrate buffer (pH 5.0) for acid invertase and 100 mM HEPES-NaOH (pH 7.5) for alkaline invertase. The reaction mixtures were incubated for 45 min at 37 °C and the glucose and fructose released were determined with dinitrosalicylic acid.

### 3.11. Antioxidant Enzyme Activity

#### 3.11.1. Preparation of Enzyme Extracts

To determine the enzymatic activities of the antioxidant proteins, a crude enzyme extracts was prepared by homogenizing 500 mg of leaf tissue in extraction buffer containing 0.5% Triton X-100 and 1% polyvinylpyrrolidone in 100 mM potassium phosphate buffer (pH 7.0) using a chilled mortar and pestle. The homogenate was centrifuged at 15,000 rpm for 20 min at 4 °C. The supernatant was used for the enzymatic assays desribed below.

#### 3.11.2. Assay of Ascorbate Peroxidase (APX) Activity

Ascorbate peroxidase activity (APX, EC 1.11.1.11) was determined spectophotometrically by a decrease in the absorbance at 265 nm using the method of Nakano and Asada [96]. The reaction mixture contained 50 mM potassium phosphate buffer pH 7.0, 5 mM ascorbate, 0.5 mM H_2_O_2_ and enzyme extract.

#### 3.11.3. Assay of Catalase (Cat) Activity

Catalase activity (CAT; EC 1.11.1.6) was determined by consumption of H_2_O_2_ using the method of Aebi [97]. The reaction mixture (3 mL) contained 50 mM potassium phosphate buffer pH 7.0, 15 mM H_2_O_2_ and 50 μL enzyme extract. The reaction was initiated by adding the H_2_O_2_. The consumption of H_2_O_2_ was monitored spectrophotometrically at 240 nm for 3 min. Enzyme activity was expressed in micromole per liter H_2_O_2_ min^−1^.

#### 3.11.4. Assay of Superoxide Dismutase (Sod) Activity

The activity of SOD (EC 1.15.1.1) was determined by measuring its ability to inhibit the photoreduction of nitro blue tetrazolium (NBT) according to the methods of Giannopolitis and Ries [98]. The reaction solution (3 mL) contained 50 μmol NBT, 1.3 riboflavin, 13 mmol methionine, 75 nmol EDTA, 50 mmol phosphate buffer (pH 7.8) and 50 μL enzyme extract. The reaction solution was irradiated under a bank of fluorescent light at 75 μmol/m^2^/s for 15 min. The absorbance at 560 was read against a blank (non-irradiated reaction solution). One unit of SOD activity was defined as the amount of enzyme that inhibited 50% of NBT photoreduction.

### 3.12. Statistical Analysis

Data were analyzed using analysis of variance by SAS version 17. Mean separation test between treatments was performed using Duncan multiple range test and standard error of differences between means was calculated with the assumption that data were normally distributed and equally replicated. Correlation analysis was analyzed using SPSS version 13 using Pearson correlation methods [99–103].

## 4. Conclusions

Our results indicate that the manipulation of fertilizer, especially potassium, may be an effective method to increase the expression of secondary metabolites in *L. pumila*. Higher total flavonoids, phenolics, and ascorbic acid were demonstrated in *L. pumila* when fertilized with high potassium (270 kg K/ha). The significant positive correlations of production of total flavonoids, phenolics and ascorbic acid content with photosynthesis, stomatal conductance and apparent quantum yield indicate the occurrence of the up-regulation of production of CBSM under enhanced photosynthetic capacity under high potassium fertilization. It also observed that the antioxidant enzymes (APX, CAT, SOD) have a significant negative relationship with total phenolics and flavonoids that indicate the reduction of antioxidant enzyme activity under high potassium fertilization concomitantly enhances the production of secondary metabolites under this condition. The increase in the production of *L. pumila* secondary metabolites under high potassium fertilization might be due to enhancement of PAL activity that was shown to have a significant positive correlation with plant secondary metabolites. Under high potassium fertilization, it was also noted that the production of soluble protein and invertase activity content were increase that were followed by the increased production of *L. pumila* secondary metabolites.

## Figures and Tables

**Table 1:** Impact of potassium levels on total phenolics, flavonoids and ascorbic acid production in different parts of *L. pumila* Benth.

Plant parts	Potassium levels (Kg K/ha)	Total phenolics (mg g^−1^ gallic acid dry weight)	Total flavonoid (mg g^−1^ rutin dry weight)	Ascorbic acid (mg g^−1^ dry weight)
	0	1.22 ± 0.44 ^d^	0.68 ± 0.05 ^d^	0.030 ± 0.006 ^d^
Leaves	90	1.40 ± 0.01 ^c^	0.75 ± 0.23 ^c^	0.047 ± 0.001 ^c^
	180	1.65 ± 0.15 ^b^	0.82 ± 0.12 ^b^	0.062 ± 0.003 ^b^
	270	1.82 ± 0.61 ^a^	0.94 ± 0.25 ^a^	0.083 ± 0.003 ^a^
	0	0.62 ± 0.23 ^d^	0.35 ± 0.15 ^c^	0.018 ± 0.004 ^d^
Stems	90	0.70 ± 0.44 ^c^	0.42 ± 0.32 ^b^	0.046 ± 0.005 ^c^
	180	0.85 ± 0.24 ^b^	0.59 ± 0.08 ^a^	0.057 ± 0.008 ^b^
	270	0.96 ± 0.34 ^a^	0.61 ± 0.14 ^a^	0.074 ± 0.004 ^a^
	0	0.40 ± 0.11 ^d^	0.18 ± 0.05 ^c^	0.010 ± 0.003 ^d^
Roots	90	0.42 ± 0.12 ^c^	0.21 ± 0.31 ^b^	0.040 ± 0.002 ^c^
	180	0.58 ± 0.05 ^b^	0.25 ± 0.13 ^b^	0.051 ± 0.001 ^b^
	270	0.62 ± 0.21 ^a^	0.41 ± 0.03 ^a^	0.073 ± 0.001 ^a^

All analyses are mean ± standard error of mean (SEM). *N* = 40. Means not sharing a common letter within a column were significantly different at *p* ≤ 0.05.

**Table 2:** Pearson correlation parameters during the experiment.

Parameters	1	2	3	4	5	6	7	8	9	10	11	12	13	14	15	16	17	18
1. Phenolics	1.000																	
2. Flavonoids	0.987 *	1.000																
3. Ascorbic acid	0.902 *	0.765 *	1.000															
4. Soluble sugar	0.879 *	0.887 *	0.809 *	1.000														
5. Starch	0.884 *	0.873 *	0.805 *	0.879 *	1.000													
6. TNC	0.985 *	0.812 *	0.765 *	0.806*	0.777 *	1.000												
7. Protein	0.678 *	0.879 *	0.768 *	0.777	0.677	0.776 *	1.000											
8. PAL	0.945 *	0.987 *	0.915 *	0.954*	0.876 *	0.778 *	0.877 *	1.000										
9. Photosynthesis	0.998 *	0.897 *	0.789 *	0.776 *	0.778 *	0.887 *	0.556	0.778 *	1.000									
10. gs	0.956 *	0.804*	0.667	0.765 *	0.667	0.885 *	0.446	0.667 *	0.987 *	1.000								
11. C_i_	0.887 *	0.702 *	0.556	0.644	0.554	0.345	0.334	0.443	0.908 *	0.667 *	1.000							
12. Rd	−0.567	−0.445	−0.567	−0.563	0.445	−0.065	−0.084	0.443	0.098	0.098	0.098	1.000						
13. APY	0.786 *	0.654	0.576	0.445	0.334	0.087	0.087	0.223	0.765 *	0.657 *	0.045	0.078	1.000					
14. Acid invertase	0.987 *	0.879 *	0.876 *	0.778 *	0.767 *	0.764 *	0.786 *	0.889 *	0.879 *	0.765 *	0.776 *	0.067	0.010	1.000				
15. Alkaline invertase	0.912 *	0.889 *	0.776 *	0.887 *	0.879 *	0.776 *	0.665 *	0.897 *	0.986 *	0.778 *	0.786 *	0.086	0.010	0.987 *	1.000			
16. APX	−0.786 *	−0.879 *	0.067	0.045	0.213	0.012	0.221	0.312	0.124	0.432	0.341	0.311	0.080	0.123	0.034	1.000		
17. CAT	−0.675 *	−0.897 *	0.456	0.043	0.321	0.123	0.123	0.034	0.012	0.123	0.113	0.123	0.070	0.234	0.076	0.021	1.000	
18. SOD	−0.876 *	−0.961 *	0.452	0.021	0.111	0.211	0.321	0.112	0.345	0.111	0.213	0.051	0.330	0.211	0.765	0.065	0.231	1.000

* significant at *p* ≤ 0.05 or *p* ≤ 0.01.

TNC = Total non structural carbohydrate; PAL = phenylalanine ammonia-lyase activity; gs = stomatal conductance; C_i_ = intercellular CO_2_; R_d_ = Dark respiration rate; APY = Apparent quantum yield; APX = ascorbate peroxidase; CAT = catalase; SOD = superodixe dismutase.

**Table 3:** Impact of potassium levels on soluble sugar, starch and total non structural carbohydrate production in different parts of *L. pumila* Benth.

Plant parts	Potassium levels (Kg K/ha)	Soluble sugar (mg g^−1^ sucrose dry weight)	Starch (mg g^−1^ glucose dry weight)	Total non structural carbohydrate (mg g^−1^ dry weight)
	0	48.23 ± 0.23 ^c^	87.11 ± 0.24 ^d^	133.42 ± 11.34 ^d^
Leaves	90	42.11 ± 2.46 ^d^	91.17 ± 3.12 ^c^	140.21 ± 12.31 ^c^
	180	52.31 ± 0.90 ^b^	96.11 ± 1.66^b^	148.11 ± 10.23 ^b^
	270	54.13 ± 3.42 ^a^	99.04 ± 1.13 ^a^	152.43 ± 10.14 ^a^
	0	55.13 ± 3.25 ^d^	78.34 ± 0.48 ^c^	117.31 ± 10.01 ^d^
Stems	90	42.22 ± 2.16 ^c^	80.12 ± 2.14 ^b^	121.40 ± 8.35 ^c^
	180	43.11 ± 0.36 ^b^	80.92 ± 2.21 ^b^	122.41 ± 7.19 ^b^
	270	47.81 ± 2.47 ^a^	86.01 ± 1.43 ^a^	134.61 ± 9.02 ^a^
	0	29.61 ± 2.12 ^d^	52.21 ± 1.13 ^c^	82.21 ± 6.89 ^d^
Roots	90	34.21 ± 3.13 ^c^	57.21 ± 1.23 ^d^	93.21 ± 4.16 ^c^
	180	35.42 ± 0.73 ^b^	60.17 ± 2.07 ^b^	96.27 ± 12.14 ^b^
	270	37.22 ± 1.07 ^a^	77.48 ± 4.13 ^a^	115.18 ± 7.35 ^a^

All analyses are mean ± standard error of mean (SEM). *N* = 40. Means not sharing a common letter within a column were significantly different at *p* ≤ 0.05.

**Table 4:** Phenylalanine ammonia-lyase activity and soluble protein content in leaves of *Labisia pumila* under different potassium fertilization.

Potassium levels (Kg K/ha)	PAL activity (nM transcinnamic mg^−1^ protein^−1^ hour^−1^)	Soluble Protein (mg g^−1^ fresh weight)
0	8.24 ± 0.23 ^d^	1.17 ± 0.34 ^d^
90	19.28 ± 0.05 ^c^	3.28 ± 0.08 ^c^
180	25.61 ± 0.34 ^b^	6.97 ± 0.11 ^b^
270	37.28 ± 2.11 ^a^	12.38 ± 0.21 ^a^

All analyses are mean ± standard error of mean (SEM). *N* = 40. Means not sharing a common letter within a column were significantly different at *p* ≤ 0.05.

**Table 5:** Net photosynthesis (A), stomatal conductance (g_s_), Intercellular CO_2_, dark respiration (R_d_) and apparent quantum yield (ξ) of *L. pumila* under different potassium fertilization regimes.

Parameters	0 kg K/ha	90 kg K/ha	180 kg K/ha	270 kg K/ha
Net photosynthesis, A (μmol m^−2^ s^−1^)	3.45 ± 0.24 ^d^	6.75 ± 0.31 ^c^	8.45 ± 0.04 ^b^	12.72 ± 0.12 ^a^
Stomatal conductance, g_s_ (mmol m^−2^ s^−1^)	12.32 ± 0.12 ^d^	19.21 ± 0.25 ^c^	28.72 ± 0.31 ^b^	40.25 ± 0.14^a^
Intercellular CO_2_, C_i_ (μmol m^−2^ s^−1^)	250 ± 10.32 ^d^	292 ± 23.34 ^c^	312 ± 25.11 ^c^	325 ± 30.12 ^a^
Dark respiration, R_d_ (μmol m^−2^ s^−1^)	8.24 ± 3.14 ^a^	6.23 ± 3.93 ^b^	4.11 ± 1.07 ^c^	2.17 ± 0.91 ^d^
Apparent quantum yield, AQY (μmol m^−2^ s^−1^)	0.032 ± 0.001 ^d^	0.043 ± 0.005 ^c^	0.078 ± 0.003 ^b^	0.097 ± 0.004 ^a^

All analyses are mean ± standard error of mean (SEM). *N* = 40. Means not sharing a common letter within a column were significantly different at *p* ≤ 0.05.

**Table 6:** Ascorbate peroxidase (APX), catalase (CAT) and superoxide dismutase (SOD) of *L. pumila* under different potassium fertilization regimes.

Potassium levels (Kg K/ha)	Ascorbate peroxidase activity (APX; mg protein^−1^ min^−1^)	Catalase activity (CAT; μmol mg protein^−1^ min^−1^)	Superoxide dismutase activity (SOD; mg protein^−1^ min^−1^)
0	10.72 ± 0.21 ^a^	171.24 ± 2.44 ^a^	230.45 ± 2.36 ^a^
90	8.74 ± 0.76 ^b^	160.32 ± 1.23 ^b^	196.72 ± 1.34 ^b^
180	6.23 ± 0.45 ^c^	110.34 ± 0.12 ^c^	172.51 ± 1.87 ^c^
270	3.44 ± 1.32 ^d^	101.24 ± 0.23 ^d^	136.24 ± 2.23 ^d^

All analyses are mean ± standard error of mean (SEM). *N* = 40. Means not sharing a common letter within a column were significantly different at *p* ≤ 0.05.

**Table 7:** The soiless medium properties.

Characteristics	Results
pH	5.8
EC (ds/m)	0.6
Nitrogen (%)	1.0%
Phosphorous (%)	0.9%
Potassium (%)	1.6%
Sulfur (%)	0.7%
Calcium (%)	0.8%
Magnesium (%)	0.4%
